# Downregulation of DNA methyltransferase-3a ameliorates the osteogenic differentiation ability of adipose-derived stem cells in diabetic osteoporosis via Wnt/β-catenin signaling pathway

**DOI:** 10.1186/s13287-022-03088-4

**Published:** 2022-08-04

**Authors:** Maorui Zhang, Yujin Gao, Qing Li, Huayue Cao, Jianghua Yang, Xiaoxiao Cai, Jingang Xiao

**Affiliations:** 1 Department of Oral Implantology, The Affiliated Stomatological Hospital of Southwest Medical University, Luzhou, 646000 China; 2 State Key Laboratory of Oral Diseases, West China Hospital of Stomatology, Sichuan University, Chengdu, 610041 China; 3 Department of Oral and Maxillofacial Surgery, The Affiliated Hospital of Southwest Medical University, Luzhou, 646000 China; 4 Luzhou Key Laboratory of Oral & Maxillofacial Reconstruction and Regeneration, The Affiliated Stomatological Hospital of Southwest Medical University, Luzhou, 646000 China

**Keywords:** DNA methyltransferase-3a, Diabetic osteoporosis, Adipose-derived stem cells, Osteogenic differentiation, Wnt/β-catenin signaling pathway

## Abstract

**Background:**

Diabetes-related osteoporosis (DOP) is a chronic disease caused by the high glucose environment that induces a metabolic disorder of osteocytes and osteoblast-associated mesenchymal stem cells. The processes of bone defect repair and regeneration become extremely difficult with DOP. Adipose-derived stem cells (ASCs), as seed cells in bone tissue engineering technology, provide a promising therapeutic approach for bone regeneration in DOP patients. The osteogenic ability of ASCs is lower in a DOP model than that of control ASCs. DNA methylation, as a mechanism of epigenetic regulation, may be involved in DNA methylation of various genes, thereby participating in biological behaviors of various cells. Emerging evidence suggests that increased DNA methylation levels are associated with activation of Wnt/β-catenin signaling pathway. The purpose of this study was to investigate the influence of the diabetic environment on the osteogenic potential of ASCs, to explore the role of DNA methylation on osteogenic differentiation of DOP-ASCs via Wnt/β-catenin signaling pathway, and to improve the osteogenic differentiation ability of ASCs with DOP.

**Methods:**

DOP-ASCs and control ASCs were isolated from DOP C57BL/6 and control mice, respectively. The multipotency of DOP-ASCs was confirmed by Alizarin Red-S, Oil Red-O, and Alcian blue staining. Real-time polymerase chain reaction (RT-PCR), immunofluorescence, and western blotting were used to analyze changes in markers of osteogenic differentiation, DNA methylation, and Wnt/β-catenin signaling. Alizarin Red-S staining was also used to confirm changes in the osteogenic ability. DNMT small interfering RNA (siRNA), shRNA-Dnmt3a, and LVRNA-Dnmt3a were used to assess the role of Dnmt3a in osteogenic differentiation of control ASCs and DOP-ASCs. Micro-computed tomography, hematoxylin and eosin staining, and Masson staining were used to analyze changes in the osteogenic capability while downregulating Dnmt3a with lentivirus in DOP mice in vivo.

**Results:**

The proliferative ability of DOP-ASCs was lower than that of control ASCs. DOP-ASCs showed a decrease in osteogenic differentiation capacity, lower Wnt/β-catenin signaling pathway activity, and a higher level of Dnmt3a than control ASCs. When Dnmt3a was downregulated by siRNA and shRNA, osteogenic-related factors Runt-related transcription factor 2 and osteopontin, and activity of Wnt/β-catenin signaling pathway were increased, which rescued the poor osteogenic potential of DOP-ASCs. When Dnmt3a was upregulated by LVRNA-Dnmt3a, the osteogenic ability was inhibited. The same results were obtained in vivo.

**Conclusions:**

Dnmt3a silencing rescues the negative effects of DOP on ASCs and provides a possible approach for bone tissue regeneration in patients with diabetic osteoporosis.

## Background

Diabetic osteoporosis (DOP) is a systemic metabolic bone disease that involves bone mass reduction, destruction of the bone tissue microstructure, and prone fractures [1, 2]. The high glucose environment and metabolic disorders caused by diabetes disrupt physiological activities such as cell growth, proliferation, and differentiation [3]. Glucose metabolism disorders break the balance between osteogenesis and osteoclast processes, which reduces the numbers of osteoblasts and mesenchymal stem cells (MSCs), and activation of osteoclasts [4, 5]. The imbalance of bone metabolism also reduces the bone differentiation ability and makes it difficult to repair bone tissue and regenerate bone [6].

In recent years, bone tissue engineering technology has provided a new approach for regeneration of bone defects. Adult stem cells are a major element of bone regeneration and have become a major research topic [7–9]. Adipose-derived mesenchymal stem cells (ASCs), as a type of MSC, have a multi-directional differentiation potential for osteogenic, cartilage, and adipose cell lineages [10–12]. They are widely used in studies of bone defect repair and regeneration, and have positive application prospects. However, the proliferation and differentiation of ASCs may be affected in the diabetic environment. Therefore, it is worth exploring whether DOP-ASCs have a normal osteogenic differentiation ability.

DNA methylation is a mechanism of epigenetic regulation. It is generally believed that the hypermethylation status of DNA sequences is related to inhibition of gene expression [13, 14]. There are three kinds of DNA methyltransferases (DNMTs) in animals, namely DNMT1, DNMT3a, and DNMT3b [15–17]. Studies have shown that DNMT3a is essential for establishment of mammalian DNA methylation during development [18, 19]. Scholars believe that the increased expression of DNMT3a regulates the increased DNA methylation level [19, 20]. Under catalysis mediated by DNMTs, the cytosines of two nucleotides of CG in DNA are selectively conjugated with methyl groups to form 5-methylcytosine (5-MC). The occurrence of various skeletal diseases, which include osteoporosis and osteoarthritis, is closely related to impaired DNA methylation in stem cells [10, 13, 21].

The canonical Wnt pathway is activated when β-catenin transfers to the nucleus and binds to TCF/LEF in the nucleus to regulate target genes [22]. β-catenin and LEF1 may reflect the status of Wnt/β-catenin pathway [23, 24]. Emerging evidence indicates that increased DNA methylation levels are associated with activation of Wnt/β-catenin pathway [25–28]. Liu T et al. [26] reported that miR708-5p inhibits the expression of Dnmt3a, resulting in the reduced global DNA methylation and, preventing β-catenin nuclear transport, thereby inhibiting Wnt/β-catenin signaling pathway. Exploring the role of DNA methylation in osteogenic differentiation of DOP-ASCs via Wnt/β-catenin signaling is not only conducive to elucidate the mechanism of DOP, but also to develop bone tissue engineering.

In our previous study, we found that advanced glycation end products inhibit the osteogenic differentiation ability of normal ASCs with a high level of DNA methylation [5]. This suggested that DNA methylation is a cause of the decline in the osteogenic differentiation ability of DOP-ASCs in the diabetic environment. In this study, we isolated ASCs from control and DOP C57BL/6 mice and compared their osteogenic differentiation potentials. Moreover, we investigated whether DNA methylation inhibits the osteogenic differentiation potential of DOP-ASCs by modulating Wnt/β-catenin signaling pathways.

## Methods

### Isolation and culture of ASCs and DOP-ASCs

All procedures that involved animals were reviewed and approved by the Southwest Medical University Ethical Committee. Anesthesia and animal care were implemented by following the guidelines for the Care and Use of Laboratory Animals (Ministry of Science and Technology of China, 2006). Adipose tissue in the inguinal region was collected from C57BL/6 DOP and control mice under sterile conditions. The adipose tissue was cut finely and fragments were seeded in 25-cm^2^ culture flasks (Corning Inc., NY) and cultured in alpha-modified Eagle’s medium (α-MEM, Hyclone, USA) supplemented with 10% fetal bovine serum (FBS, Hyclone) and 1% penicillin/streptomycin (Hyclone) at 37 °C with 5% CO_2_. The medium was changed every 3 days. Adherent cells were cultured and non-adherent cells were removed.

DOP-ASCs were passaged three times to obtain relatively pure ASCs. Osteogenic, adipogenic, and cartilage media (Cyagen, USA) were used to define the multipotential differentiation capacity of DOP-ASCs. DOP-ASCs (5 × 10^4^ cells) were seeded in a 6-well plate for osteogenic induction. DOP-ASCs (1 × 10^5^ cells) were also seeded for adipogenic induction. All cells were cultured for 21 days. Then, the cells were washed three times with PBS and fixed with 4% paraformaldehyde for 1 h. Alizarin Red-S (osteogenic dye) and 0.3% Oil Red-O (adipogenic dye) were used to stain mineralized nodules and lipid droplets, respectively, for 30 min. The stained cells were imaged under an inverted phase contrast microscope (Nikon, Japan). For cartilage induction, DOP-ASCs (2.5 × 10^5^ cells) were centrifuged and cell aggregates were cultured in cartilage medium. After 21 days, the cell aggregates were washed three times with PBS and fixed with 4% paraformaldehyde. The cartilage pellets were imaged under a stereo fluorescence microscope (Carl Zeiss Microscopy, Germany). Then, they were embedded in paraffin and sections were stained with Alcian blue. Cartilage matrix was imaged under an optical microscope (Nikon).

### Proliferation assay

A Cell Counting Kit-8 (CCK-8) assay (Sigma-Aldrich, St Louis, Missouri, USA) and xCelligence system for real-time cellular analysis (RTCA) (Roche Diagnostics GmbH, Basel, Switzerland) were used to assess cell proliferation. For the CCK-8 assay, cells were seeded in 96-well plates (Corning Inc.) at a density of 3 × 10^3^ cells per well and cultured in α-MEM with 10% FBS for 5 days. A BioTek ELX800 (Bio-Tek, USA) was used to measure absorbance at 450 nm. For RTCA, cells were seeded in 96-well E-plates (Roche Diagnostics GmbH) at 3 × 10^3^ cells per well. Cell proliferation in the RTCA SP xCelligence system was monitored in real-time as the impedance value over 5 days. Data were analyzed by the provided RTCA software.

### Alizarin red-S staining

Mineralized nodule formation in ASCs was stained by Alizarin Red-S (Cyagen). DOP-ASCs and control ASCs (5 × 10^4^ cells) in 6-well plates were treated with osteogenic medium for 21 days. Cells were then washed with PBS three times, fixed in 4% paraformaldehyde for 1 h, and stained with Alizarin Red-S for 30 min.

### Real-time polymerase chain reaction (RT-PCR)

Total RNA was extracted using a Total mRNA Extraction Kit (Takara Bio, Japan). cDNA was synthesized by reverse transcription using a Prime Script Reverse Transcription Reagent Kit (Takara Bio). Then, RT-PCR was conducted to measure the gene expression of Runt-related transcription factor 2 (*Runx2*), osteopontin (*Opn*), DNA methyltransferase 1/3a/3b (*Dnmt1/3a/3b*), *β-catenin*, and lymphoid enhancer-binding factor-1 (*Lef1*). Primer sequences are shown in Table 1. Samples were analyzed using a SYBR Premix ExTaq kit (Takara Bio), following the standard procedure, in an ABI 7900 system (Applied Biosystems, USA), which included melting curve analysis and obtaining CT values. The results were normalized to *Gapdh* CT values and the 2^−∆∆Ct^ method was used to calculate gene expression.

**Table 1:** Primer sequences for RT-PCR

Genes		Sequence (5′ → 3′)
*Gapdh*	Forward	GGTGAAGGTCGGTGTGAACG
	Reverse	CTCGCTCCTGGAAGATGGTG
*Runx2*	Forward	CCGAACTGGTCCGCACCGAC
	Reverse	CTTGAAGGCCACGGGCAGGG
*Opn*	Forward	GGATTCTGTGGACTCGGATG
	Reverse	CGACTGTAGGGACGATTGGA
*Dnmt1*	Forward	CCGAACTGGTCCGCACCGAC
	Reverse	CTTGAAGGCCACGGGCAGGG
*Dnmt3a*	Forward	GAGGGAACTGAGACCCCAC
	Reverse	CTGGAAGGTGAGTCTTGGCA
*Dnmt3b*	Forward	AGCGGGTATGAGGAGTGCAT
	Reverse	GGGAGCATCCTTCGTGTCTG
*β-Catenin*	Forward	AAGTTCTTGGCTATTACGACA
	Reverse	ACAGCACCTTCAGCACTCT
*Lef1*	Forward	ACAGATCACCCCACCTTCTTG
	Reverse	TGATGGGAAAACCTGGACAT

### Western blot assay

A Total Protein Extraction Kit (Keygen Biotech, China) was used to extract total cellular proteins. A bicinchoninic acid protein assay kit (Thermo Fisher Scientific, MA, USA) was used to measure the protein concentration. Proteins were separated by 10% (v/v) sodium dodecyl sulfate–polyacrylamide gel electrophoresis and then transferred onto a polyvinylidene difluoride membrane at 200 mA for 1 h. Tris-buffered saline with 0.05% (v/v) Tween-20 (TBST) was used to dissolve dry skimmed milk (Keygen Biotech). PVDF membranes were blocked with 5% dry skimmed milk for 1 h and then incubated with antibodies against GAPDH (ab181602), DNMT3a (ab188470), DNMT3b (ab79822), and OPN (ab91655) (Abcam, UK), RUNX2 (12556 s), DNMT1 (5032S), β-catenin (D10A8), or LEF1 (2230p) (Cell Signaling Technology, USA) for 1 day at 4 °C. Then, PVDF membranes were washed three times with TBST and incubated with a goat anti-rabbit secondary antibody (Beyotime, Shanghai, China) for 1 h. They were then washed again with TBST and developed with an enhanced chemiluminescence detection system (Bio-Rad, USA).

### Immunofluorescence staining

Cells were seeded on round coverslips (Corning Inc.) and cultured for 4 days. After various treatments, the cells were carefully washed three times with PBS, fixed with 4% paraformaldehyde for 1 h, and permeabilized with 0.5% Triton X-100 for 10 min. Then, they were blocked with 5% goat serum (Beyotime) for 1 h and incubated for 1 day at 4 °C with antibodies against RUNX2, OPN, DNMT1, DNMT3a, DNMT3b, 5-MC (28692S), β-catenin, or LEF1. The next day, the samples were incubated with a fluorescent dye-conjugated secondary antibody (Beyotime) for 1 h. Nuclei were counterstained with 4ʹ6-diamidino-2-phenylindole (Beyotime) for 10 min and phalloidin (Beyotime) was used to stain microfilaments for 10 min. Cells were imaged under a laser scanning confocal microscope (Olympus, Japan).

### Transfection of small interfering RNA (siRNA)

Small interfering RNA (siRNA) that targeted *Dnmt1*, *Dnmt3a*, and *Dnmt3b* was designed and provided by GenePharma Co., Ltd (Shanghai, China). siRNA sequences are shown in Table 2. DOP-ASCs (5 × 10^4^ cells) were seeded in a 12-well plate before siRNA transfection. The transfection reagent (Lipofectamine 2000; Thermo Fisher Scientific) was diluted with Opti-MEM I Reduced Serum Medium (Hyclone) and incubated at room temperature for 5 min. The siRNA was added to the diluted Lipofectamine 2000 and gently mixed to form the siRNA-lipofectamine-Opti-MEM complex. Then, the mixture was added to cells at 1 ml per well and incubated at 37 °C with 5% CO_2_.

**Table 2:** siRNA sequences for gene silencing

siRNA		Sequence (5′ → 3′)
*Dnmt1*	Sense	CCGAAGAUCAACUCACCAATT
	Antisense	UUGGUGAGUUGAUCUUCGGTT
*Dnmt3a*	Sense	CCAUGUACCGCAAAGCCAUTT
	Antisense	AUGGCUUUGCGGUACAUGGTT
*Dnmt3b*	Sense	CCUCAAGACAAAUAGCUAUTT
	Antisense	AUAGCUAUUUGUCUUGAGGTT
*Negative control*	Sense	UUCUUCGAACGUGUCACGUTT
	Antisense	ACGUGACACGUUCGGAGAATT

### Transduction of shRNA-*Dnmt3a* and LVRNA-*Dnmt3a*

The *Dnmt3a* overexpression lentiviral vector (pLenti-EF1a-EGFP-P2A-Puro-CMV-*Dnmt3a*-3Flag) and *Dnmt3a*-silencing lentiviral vector (pLDK-CMV-EGFP-2A-Puro-U6-shRNA*Dnmt3a*) were designed and manufactured by OBiO Technology Corp., Ltd. (Shanghai, China). The oligonucleotide sequences of shRNA with Dnmt3a RNA interference targets are shown in Table 3. Various virus concentrations were used to determine the multiplicity of infection (MOI). The transduction efficiency was evaluated by analyzing the percentage of green fluorescent protein (GFP)-positive cells under a fluorescence microscope. ASCs at a density of 5 × 10^4^/ml were seeded in a 6-well plate at 2 ml per well. After 12 h of culture, the medium was replaced with a lentivirus suspension medium (MOI:80; 0.6 μg/ml puromycin; 5 μg/ml polybrene). The gene and protein expression were analyzed by RT-PCR and western blotting, respectively, after 4 days of osteogenic induction and their osteogenic ability was assessed by Alizarin Red-S staining after induction for 21 days.

**Table 3:** Dnmt3a shRNA sequences

5′		STEM	Loop	STEM	3′
sh-Dnmt3a-F	Ccgg	CCACCAGGTCAAACTCTAT	TTCAAGAGA	ATAGAGTTTGACCTGGTGG	TTTTTTg
sh-Dnmt3a-R	aattcaaaaaa	CCACCAGGTCAAACTCTAT	TCTCTTGAA	ATAGAGTTTGACCTGGTGG	
sh-NC-F	CCGG	TTCTCCGAACGTGTCACGT	TTCAAGAGA	ACGTGACACGTTCGGAGAA	TTTTTTG
sh-NC-R	AATTCAAAAAA	TTCTCCGAACGTGTCACGT	TCTCTTGAA	ACGTGACACGTTCGGAGAA	

### Analysis of DOP-ASCs seeded on BCP by scanning electron microscopy (SEM)

Before seeding DOP-ASCs, scaffolds sterilized by ultraviolet light were placed in 12-well plates. Then, 1 ml of passage 2 DOP-ASCs at a density of 5 × 10^4^/ml was seeded on the surface of BCP in each well. After culture at 37 °C with 5% CO_2_ for 3 days, samples were fixed with paraformaldehyde. After alcohol gradient dehydration, critical point drying, and spraying the cells with gold, scaffolds were observed by SEM.

### Implantation of BCP seeded with DOP-ASCs transduced with shRNA into a DOP mouse model with critically sized calvarial defects

DOP-ASCs were divided into DOP-blank, negative control, and Dnmt3a shRNA groups. DOP-ASCs infected with the silence-Dnmt3a lentivirus were cultured in osteogenic induction medium. A1-ml cell suspension (5 × 10^4^ cells/ml) was added to the surface of BCP in a 12-well plate and cultured for 48 h. Nine DOP mice received calvarial surgery to establish critically sized calvarial defect models. After anaesthetization, the DOP mice were subjected to prone fixation, skin preparation, and disinfection at the top of the skull. An incision was made along the median of the calvarium and the periosteum was bluntly separated to expose the calvarial bone surface. Then, a 4-mm diameter trephine bur was applied to drill a standardized round defect on the side of the sagittal suture. A 0.9% saline solution was used to irrigate the skull surface during drilling. Subsequently, the BCP seeded with DOP-ASCs was implanted into the skull defect area and the periosteum and dermis were sutured in position. After 8 weeks, mice were euthanized and skull specimens were obtained.

### Micro-computed tomography (Micro-CT), hematoxylin and eosin staining (HE) staining, and Masson staining

At 8 weeks, the calvarium was removed intact and fixed in freshly prepared 4% formaldehyde for 24 h at 4 °C. Micro-CT scans of skull defects were performed to observe new bone formation. Then, three-dimensional reconstructed images were analyzed. The ratio of the bone volume to total volume available in the scaffold (BV/TV) was calculated. A high ratio indicated that more bone had grown into the scaffolds. Then, tissue samples of the mouse skull defect were decalcified for HE and Masson staining. Next, the samples were dehydrated in an alcohol gradient, clarified, and embedded in paraffin for sectioning. Lastly, the sections were stained with hematoxylin and eosin and Masson trichrome.

### Statistical analysis

All experiments were repeated at least three times independently. Two group comparisons were made by the independent-samples t-test and multiple comparisons were made by one-way ANOVA with SPSS 18.0 software (SPSS Inc., Chicago, USA). *P* < 0.05 was regarded to be statistically significant.

## Results

### Cell proliferation and multipotent differentiation of DOP-ASCs

**Fig. 1:**
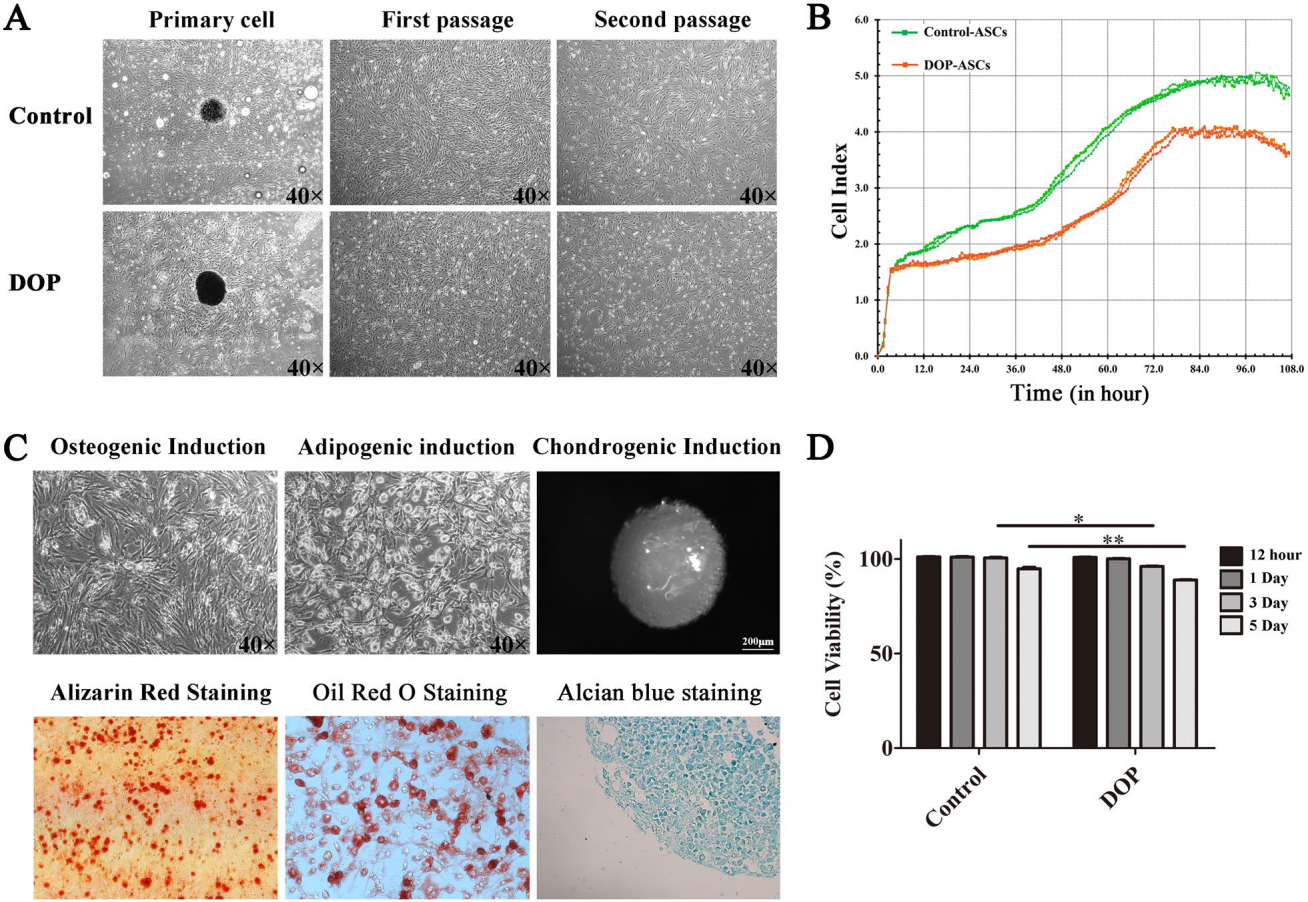
Cell proliferation and multipotent differentiation of ASCs. **A**: Normal morphology of primary, first, and second passages of control ASCs and DOP-ASCs under light microscopy. **B**, **D**: RTCA and CCK-8 assays showed that the proliferation rate of the DOP group was lower than that of the control group. **C**: Multipotent differentiation of DOP-ASCs into osteogenic cell-like, adipose-like, and cartilage-like cells

ASCs from inguinal adipose tissue were isolated and passaged three times (Fig. 1A). RTCA (Fig. 1B) and CCK-8 assays (Fig. 1D) showed that the proliferation rate of the DOP group was relatively lower than that of the control group. After culture in osteogenic and adipogenic media, the morphology of DOP-ASCs had distinctly changed to osteogenic-like in osteogenic medium and adipose-like in adipogenic medium (Fig. 1C). In cartilage medium, ASCs were aggregated to culture for 21 days and then stained with Alcian blue to indicate cartilage-like cells. The findings demonstrated the multipotency of DOP-ASCs (Fig. 1C).

### Osteogenic differentiation capacity decreases in DOP-ASCs

**Fig. 2:**
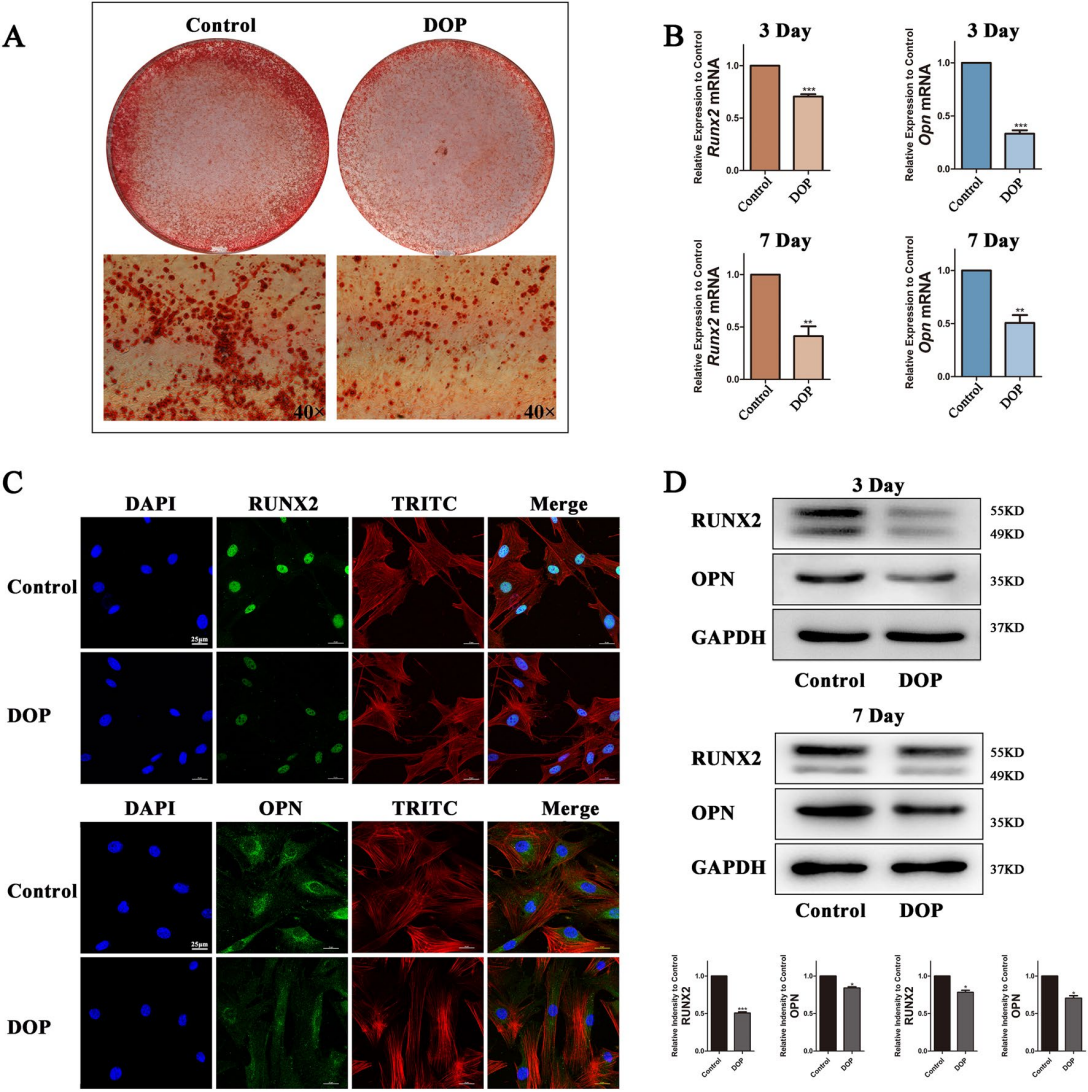
Decrease of the osteogenic differentiation capacity in DOP-ASCs. **A**: Alizarin Red-S staining of control ASCs and DOP-ASCs. **B**: mRNA levels of osteogenesis-related molecules *Runx2* and *Opn* after 3 and 7 days of osteoinduction. **C**: Immunofluorescence staining of RUX2 and OPN proteins in control ASCs and DOP-ASCs after 4 days of osteoinduction. **D**: Protein levels of osteogenesis-related molecules RUNX2 and OPN after 3 and 7 days of osteoinduction. Data represent the mean ± SD of at least three independent experiments, *P < 0.05, **P < 0.01

To investigate the osteogenic differentiation capacity, we cultured control ASCs and DOP-ASCs to analyze mineralized nodule formation as well as gene and protein expression of OPN and RUNX2. Alizarin Red-S staining showed that the degree of mineralized nodule formation was reduced in DOP-ASCs compared with control ASCs (Fig. 2A). RT-PCR showed that the mRNA levels of *Runx2* and *Opn* in DOP-ASCs were significantly lower than those in control ASCs at 3 and 7 days (Fig. 2B). The protein levels of OPN and RUNX2 were analyzed by immunofluorescence and western blotting, which showed that the fluorescence signals (Fig. 2C) and band intensities (Fig. 2D) at 4 days in DOP-ASCs were weaker compared with those in control ASCs.

### DNA methylation increases in DOP-ASCs

**Fig. 3:**
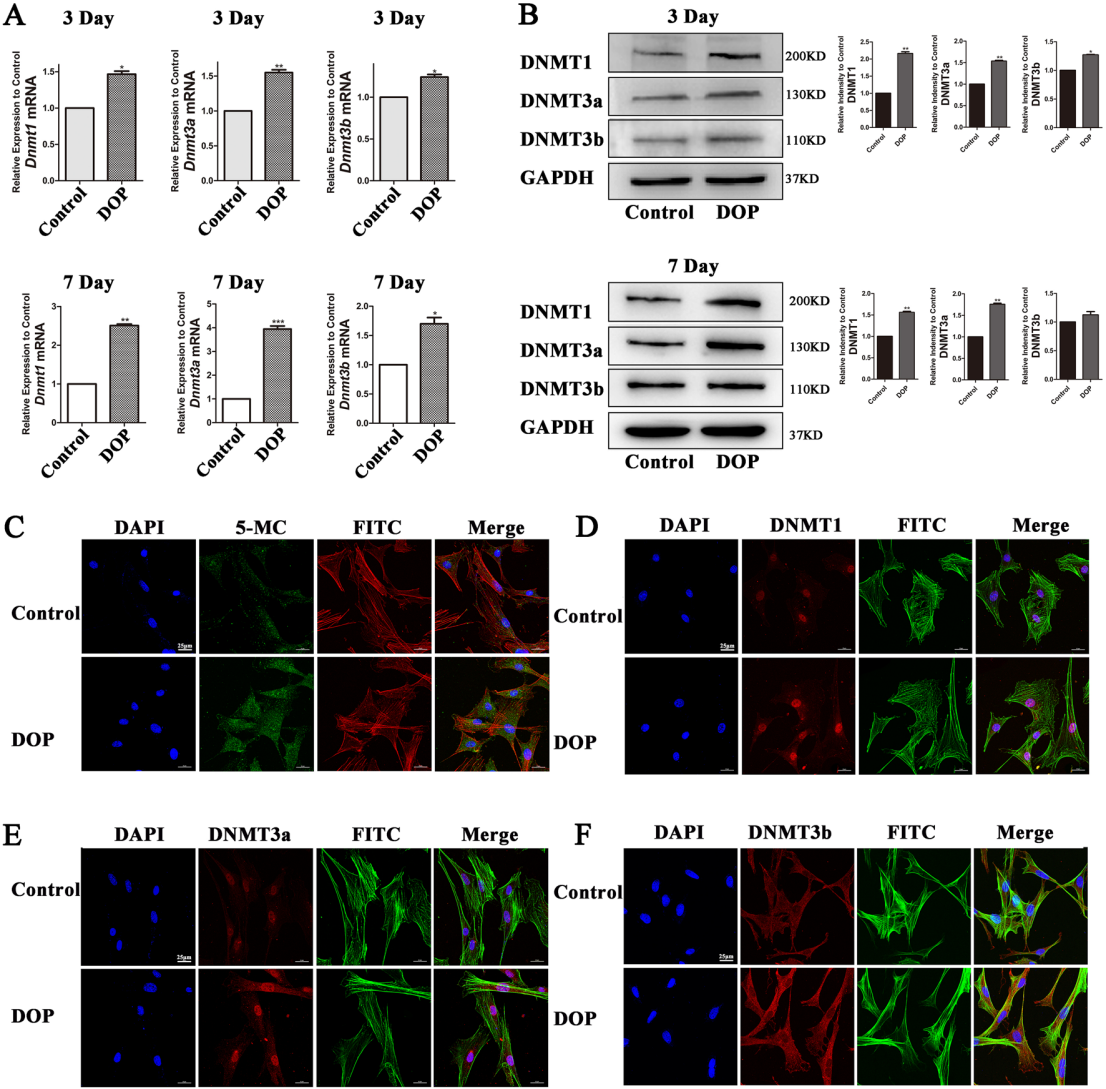
Increase of the DNA methylation level in DOP-ASCs. **A**, **B**: RT-PCR and western blot analyses showing that the expression of Dnmt1, Dnmt3a, and Dnmt3b in DOP-ASCs was increased compared with that in control ASCs. **C**, **D**: Immunofluorescence showing increases in 5-MC, DNMT1, DNMT3a, and DNMT3b after 4 days of osteoinduction. Data represent the mean ± SD of at least three independent experiments, *P < 0.05, **P < 0.01

DNMT1, DNMT3a, and DNMT3b are major enzymes in DNA methylation and 5-MC is the product of this process. We analyzed the expression of these factors by RT-PCR, western blotting, and immunofluorescence. The expression of *Dnmt1*, *Dnmt3a*, and *Dnmt3b* in DOP-ASCs increased compared with that in control ASCs (Fig. 3A, B). Immunofluorescence confirmed the increases in 5-MC, DNMT1, DNMT3a, and DNMT3b at 4 days (Fig. 3C–F).

### Wnt/β-Catenin signaling pathway is suppressed in DOP-ASCs

**Fig. 4:**
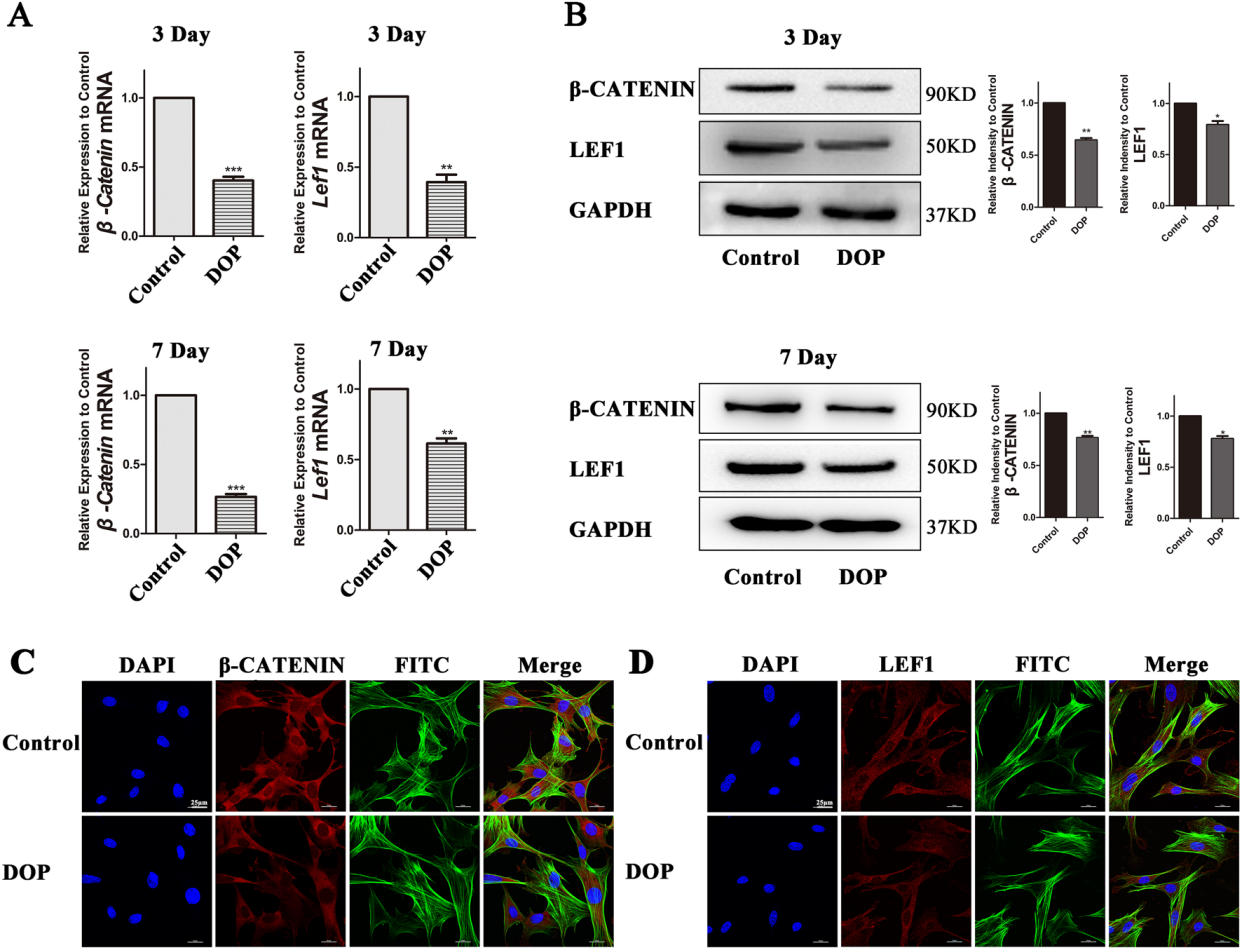
Wnt/β-Catenin signaling pathway is suppressed in DOP-ASCs. **A**, **B**: RT-PCR and western blot analyses showing that the expression of β-catenin and LEF1 was decreased compared with that in CON-ASCs. **C**, **D**: Immunofluorescence staining of β-catenin and LEF1. Data represent the mean ± SD of at least three independent experiments, *P < 0.05, **P < 0.01

The Wnt/β-Catenin signaling pathway is a major regulatory pathway in the process of osteogenic differentiation [29, 30]. Therefore, the main factors, which included *β-catenin* and *Lef1*, were detected to demonstrate the activation level of Wnt/β-Catenin signaling pathway. RT-PCR showed that the expression of *β-catenin* and *Lef1* decreased in DOP-ASCs compared with that in CON-ASCs, and the results of western blotting were consistent with those of RT-PCR (Fig. 4A, B). Immunofluorescence confirmed that the expression of β-catenin and LEF1 was low in DOP-ASCs (Fig. 4C, D).

### Inhibiting DNA methyltransferases rescues loss of the osteogenic potential in DOP-ASCs

**Fig. 5:**
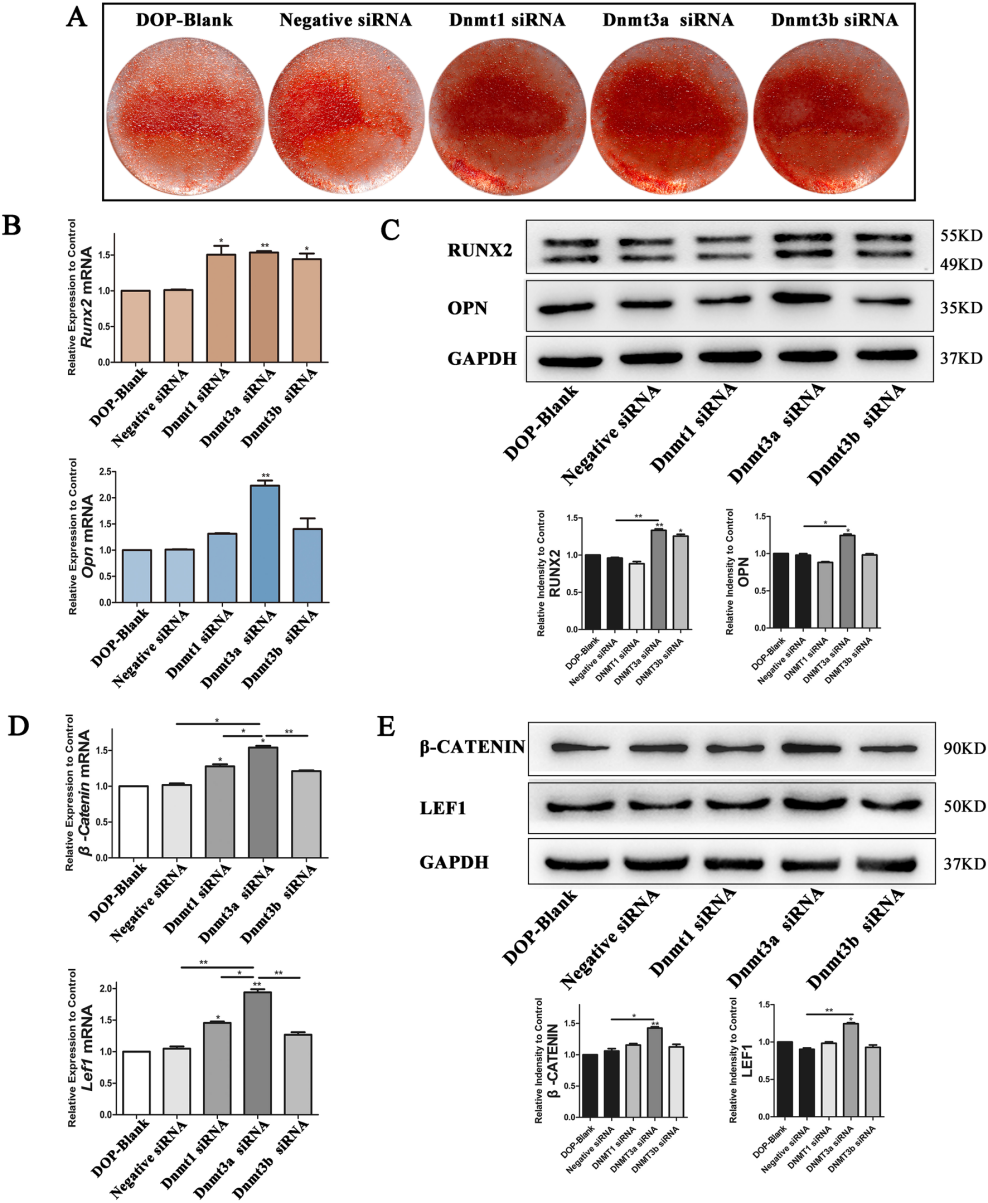
*Dnmt* siRNAs increase the osteogenic potential of DOP-ASCs. **A**: Alizarin Red-S staining showing that the formation of mineralized nodules in Dnmt3a shRNA was increased compared with that in Negative Control after *Dnmt* siRNA treatment of DOP-ASCs (osteoinduction for 21 days). **B**–**E**: mRNA and protein levels of Wnt/β-Catenin signaling pathway markers and osteogenesis-related molecules were upregulated after *Dnmt* siRNA transfection into DOP-ASCs (osteoinduction for 4 days). Data represent the mean ± SD of at least three independent experiments, *P < 0.05, **P < 0.01

The results showed that the reduced osteogenic differentiation capacity of DOP-ASCs was related to increases in DNA methylation levels and suppression of Wnt/β-Catenin signaling pathway. Next, we used siRNA to inhibit the expression of DNA methylation enzymes and explored the relationship between DNA methylation and the osteogenic differentiation ability of DOP-ASCs. After siRNA treatment, the formation of mineralized nodules was increased when the DNA methylation level was downregulated (Fig. 5A). RT-PCR and western blotting showed that RUNX2 was increased in Dnmt1-siRNA, Dnmt3a-siRNA, and Dnmt3b-siRNA groups, and OPN was particularly increased in the Dnmt3a-siRNA group (Fig. 5B, C). In terms of Wnt/β-Catenin signaling pathway, β-catenin and LEF1 were upregulated after siRNA treatment and their expression was the highest in the Dnmt3a-siRNA group compared with the other groups (Fig. 5D, E). These data suggested that downregulation of Dnmt3a inhibited osteogenic differentiation and activity of Wnt/β-Catenin signaling pathway.

### Knockdown of Dnmt3a promotes osteogenic differentiation of DOP-ASCs

**Fig. 6:**
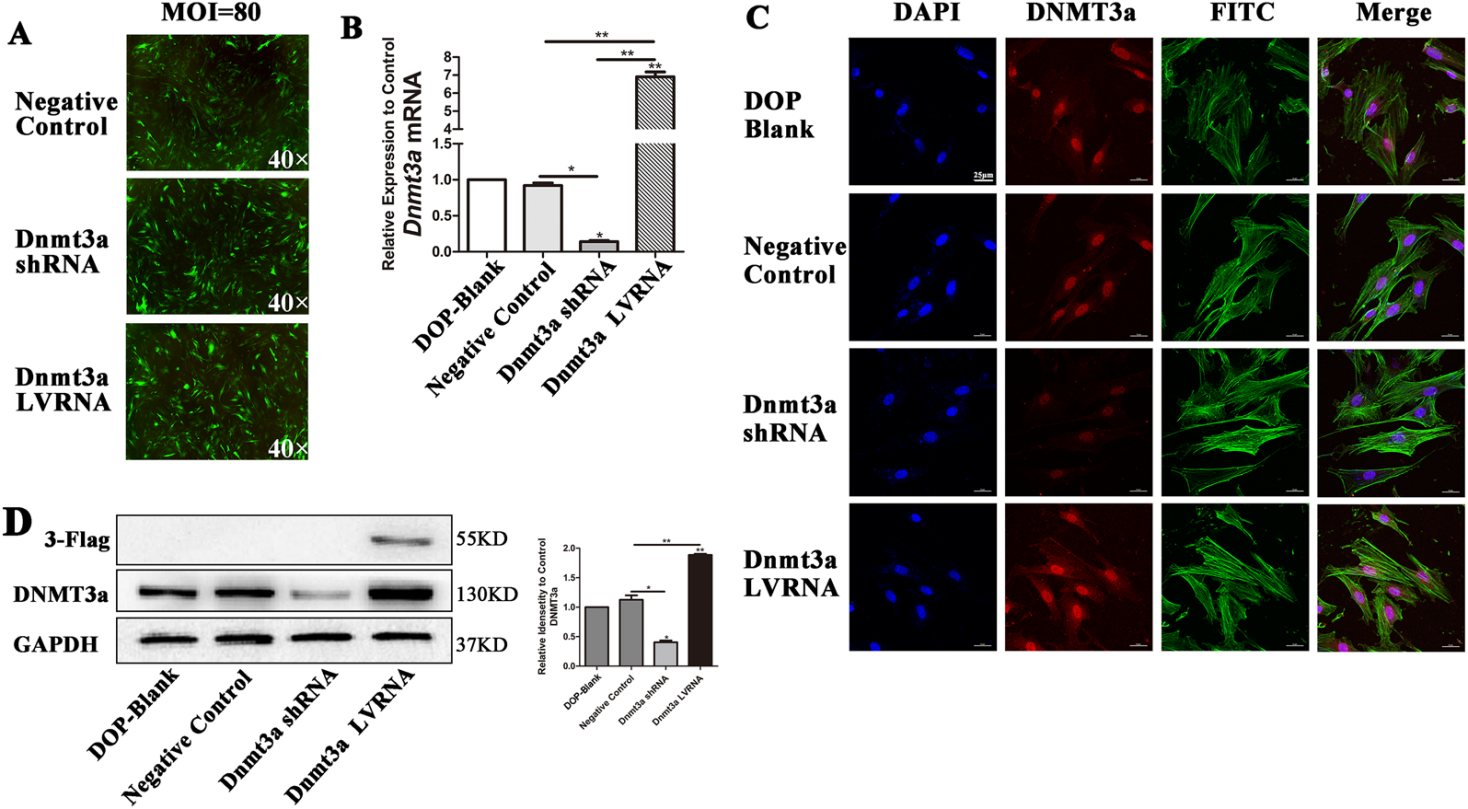
Dnmt3a shRNA and Dnmt3a LVRNA were successfully transduced into DOP-ASCs. **A**: Cells were successfully infected with lentiviruses and exhibited green fluorescence at an MOI of 80. **B**, **D**: mRNA levels of *Dnmt3a* and protein levels of 3-Flag showed that Dnmt3a was knocked down by Dnmt3a shRNA and overexpressed by Dnmt3a LVRNA. **C**: Immunofluorescence staining of Dnmt3a. Data represent the mean ± SD of at least three independent experiments, *P < 0.05, **P < 0.01

To further demonstrate the effect of Dnmt3a on osteogenic differentiation of DOP-ASCs, we used lentiviruses to knockdown or overexpress Dnmt3a in DOP-ASCs. The cells were successfully infected by the lentiviruses and showed green fluorescence at an MOI of 80 (Fig. 6A). RT-PCR and western blotting showed that Dnmt3a was successfully knocked down by Dnmt3a shRNA and overexpressed by Dnmt3a LVRNA. 3-Flag was a marker of positive overexpression (Fig. 6B, D). Immunofluorescence confirmed the differences in expression of DNMT3a among the DOP-blank group, Negative Control, Dnmt3a shRNA and Dnmt3a LVRNA. (Fig. 6C).

**Fig. 7:**
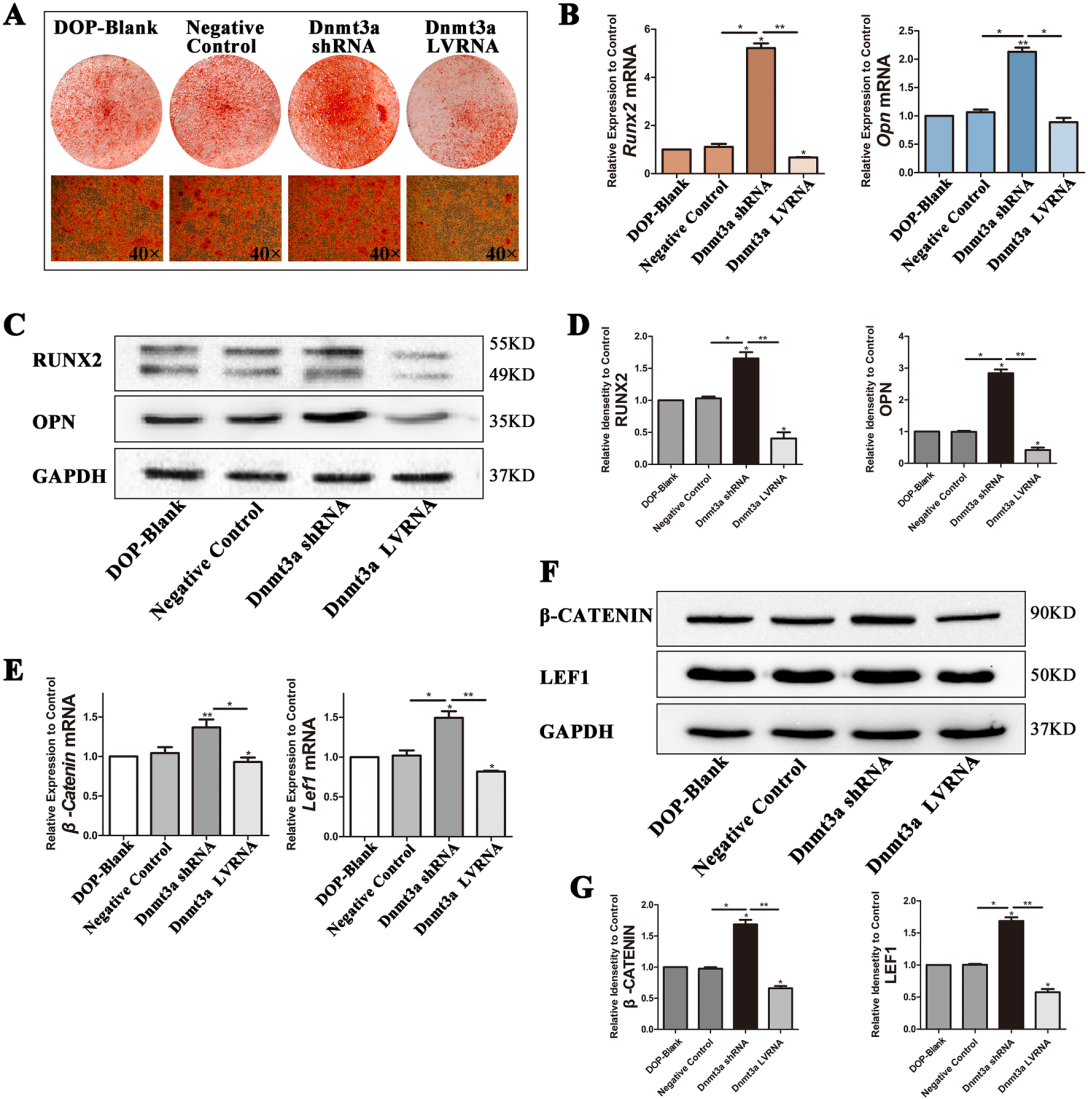
Knockdown of Dnmt3a promotes osteogenic differentiation of DOP-ASCs. **A**: Alizarin Red-S staining showing that the formation of mineralized nodules was the highest in the Dnmt3a shRNA group and the lowest in the Dnmt3a LVRNA group (osteoinduction for 21 days). **B**–**D**: mRNA and protein levels of osteogenesis-related molecules were upregulated in the Dnmt3a shRNA group compared with the other three groups (osteoinduction for 4 days). **E**–**G**: mRNA and protein levels of Wnt signaling pathway markers were recovered by knockdown of Dnmt3a. Data represent the mean ± SD of at least three independent experiments, *P < 0.05, **P < 0.01

Next, we found that the formation of mineralized nodules was the highest in the Dnmt3a shRNA group and the lowest in the Dnmt3a LVRNA group (Fig. 7A). Expression of *Opn* and *Runx2* was upregulated in the Dnmt3a shRNA group compared with the other three groups and the results of western blot assays were consistent with those of RT-PCR (Fig. 7B–D). Detection of osteogenic differentiation by Alizarin Red-S staining, RT-PCR, and western blotting showed that knockdown of Dnmt3a rescued the osteogenic differentiation capacity of DOP-ASCs. Although Dnmt3a LVRNA treatment decreased the expression of *β-catenin* and *Lef1* compared with DOP-ASC and negative control groups, the expression of these factors was recovered by Dnmt3a shRNA treatment. This suggested that the low activity of the Wnt signaling pathway in DOP-ASCs was recovered by knocking down Dnmt3a (Fig. 7E–G). Taken together, these results suggested that knockdown of Dnmt3a decreased the DNA methylation level, alleviated inhibition of Wnt by DNA methylation, and rescued the loss of the osteogenic capacity of DOP-ASCs.

### Downregulation of Dnmt3a promotes the osteogenic capacity of DOP-ASCs in vivo

**Fig. 8:**
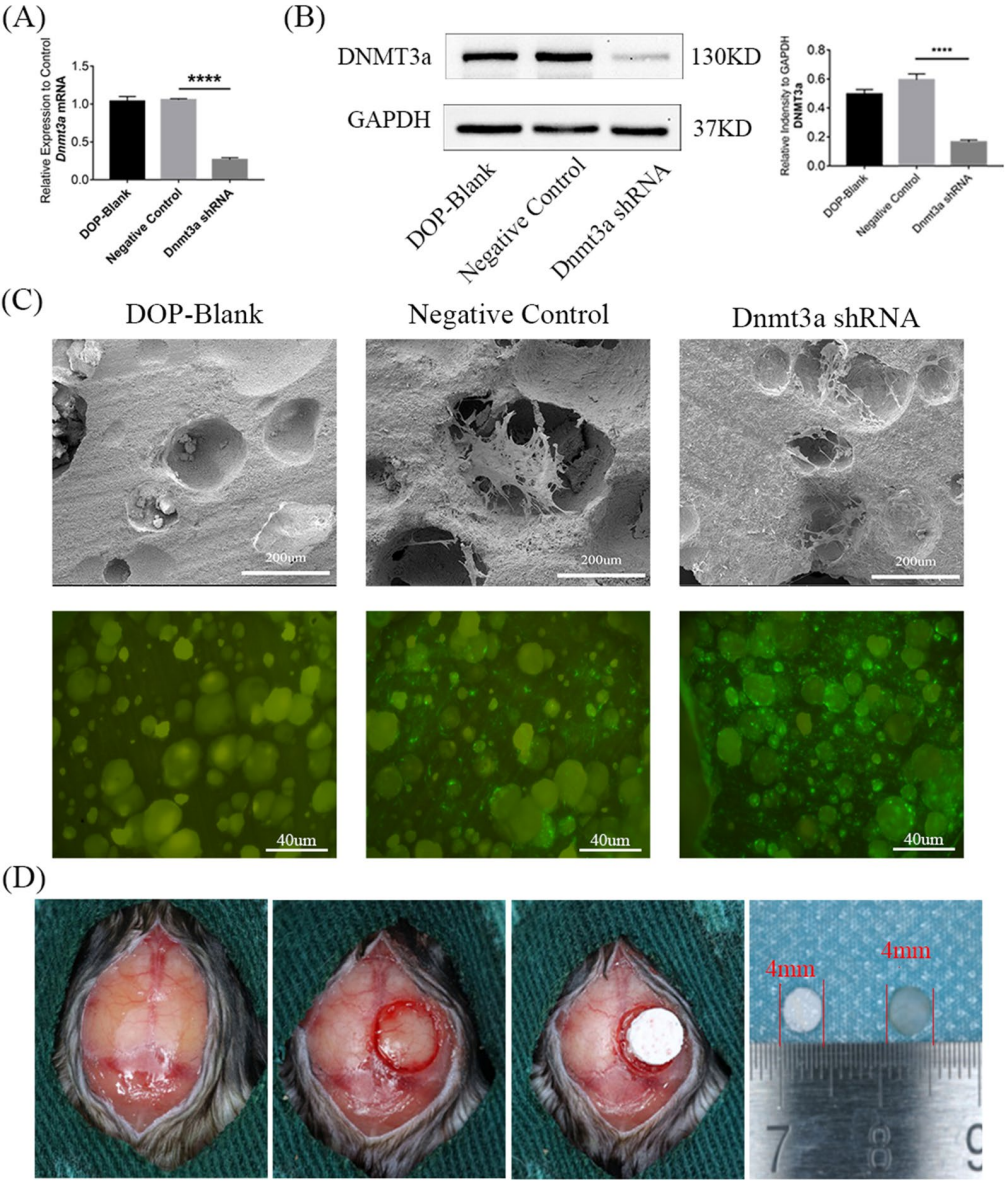
BCP was successfully implanted into the skull defect of mice. BCP seeded with transfected DOP-ASCs were implanted into critically sized calvarial defects in DOP mouse models. **A**, **B**: mRNA and protein levels of Dnmt3a were successfully knocked down by Dnmt3a shRNA (osteoinduction for 3 days). **C**: SEM and fluorescence microscopy showing that DOP-ASCs grew adherently on the surface and pores of BCP. **D**: The DOP mouse model with critically sized calvarial defects was successfully established and BCP seeded with transfected DOP-ASCs were implanted into the defect area

**Fig. 9:**
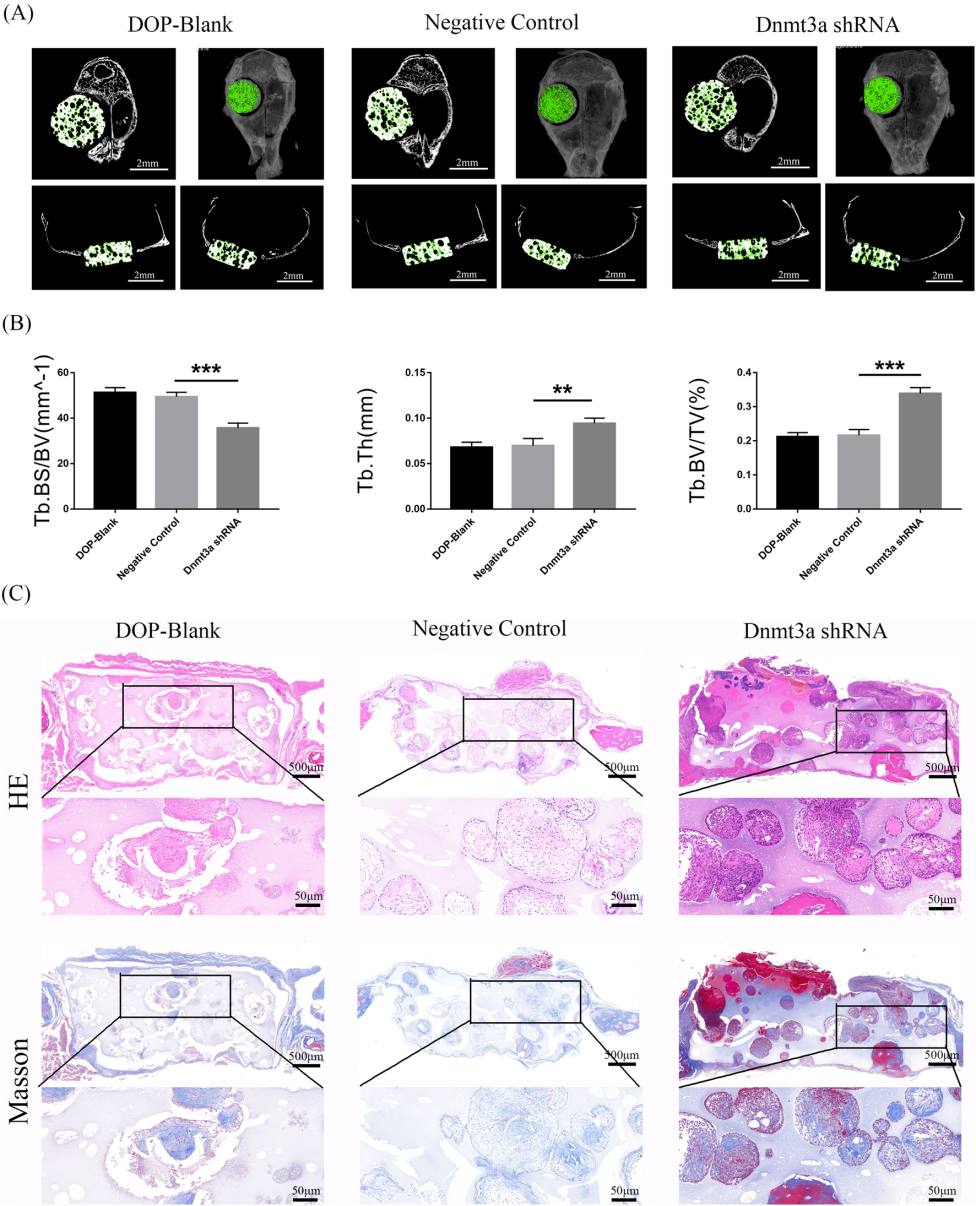
Downregulation of Dnmt3a promotes the osteogenic capacity of DOP-ASCs in vivo. **A** Micro-CT showed that the amount of new bone matrix (green) in the Dnmt3a shRNA group was significantly higher than that in DOP-ASC and negative control groups. **B** BV/TV, BS/BV, and TbTh analysis demonstrated that the osteogenic capacity was greatly increased when Dnmt3a was downregulated by shRNA in vivo. Data represent the mean ± SD of at least three independent experiments, *P < 0.05, **P < 0.01. **C**: HE and Masson staining of BCP showed that the staining degree in the Dnmt3a shRNA group was stronger than that in DOP-ASC and negative control groups

RT-PCR and western blotting showed that Dnmt3a was successfully knocked down by Dnmt3a shRNA (Fig. 8A, B). SEM and fluorescence microscopy showed that DOP-ASCs grew adherently on the surface and pores of BCP (Fig. 8C). The DOP mouse model with critically sized calvarial defects was successfully established and BCP seeded with transfected DOP-ASCs were implanted into the defect area (Fig. 8D). Eight weeks later, Micro-CT showed new bone matrix on BCP at sagittal and coronal levels. Three-dimensional reconstruction showed that the amount of new bone matrix in the Dnmt3a shRNA group was significantly larger than that in DOP-ASC and negative control groups.BV/TV, BS/BV, and TbTh analyses further demonstrated that the osteogenic capacity was greatly increased when Dnmt3a was downregulated by shRNA in vivo (Fig. 9A, B). HE and Masson staining were also used to observe the osteogenic capacity of DOP-ASCs in vivo. HE staining showed new bone matrix as red and Masson staining showed new bone matrix as blue. Both staining showed that the staining degree in the Dnmt3a shRNA group was stronger than that in DOP-ASC and negative control groups (Fig. 9C). These results suggested that knockdown of Dnmt3a rescued the loss of the osteogenic capacity of DOP-ASCs.

## Discussion

Many studies have shown that hyperglycemia and the glycolytic metabolites of diabetes decrease cell viability and proliferation, and even promote apoptosis of MSCs, which impairs osteogenic differentiation [29–33]. Heilmeier et al. found that serum miR-550a-5p inhibited the osteogenic differentiation of ASCs in postmenopausal women with type 2 diabetes [34]. Liu et al. reported that osteogenic differentiation of hPDLSCs was significantly inhibited in a high glucose environment and the levels of osteoblast-related factors expressed by cells were reduced significantly [35]. In this study, DOP-ASCs were isolated from DOP mice by the tissue block method, which had osteogenic, adipogenic, and chondrogenic differentiation abilities. However, the expression of osteogenic-related genes *Runx2* and *Opn* was downregulated in DOP-ASCs compared with control ASCs, which demonstrated inhibition of the differentiation process of DOP-ASCs to osteoblasts.

The differentiation of MSCs into osteogenic progenitor cells is regulated by various growth factors and signaling pathways [36, 37]. The Wnt/β-Catenin signaling pathway plays a major role in regulating the proliferation and differentiation of MSCs. Activation of Wnt/β-Catenin signaling pathway promotes osteogenic differentiation of ASCs [38, 39]. Moldes et al. reported that β-catenin expression was higher in 3T3-L1 precursor adipocytes and the expression level of β-catenin was significantly reduced during adipogenesis [40]. In our previous studies, after activation of Wnt/β-Catenin signaling pathway, the expression of Wnt-related signaling molecules, such as β-catenin and LEF1, was upregulated in normal ASCs, which promoted the expression of osteogenic differentiation factors such as Opn and Runx2 [5, 41]. In this study, we compared DOP-ASCs and control ASCs and demonstrated that osteogenic differentiation and Wnt/β-Catenin signaling pathway were suppressed in ASCs of DOP mice.

The high glucose environment caused by diabetes increases DNA methylation in cells, which affects their differentiation processes [42, 43]. Many studies have suggested that DNA methylation is involved in the osteogenic differentiation of stem cells [44–48]. Wang et al. reported that KDM6A promoted chondrogenic differentiation of periodontal ligament stem cells by demethylation of SOX9 [49]. Zhang et al. reported that a demethylated Runx2 gene in bone marrow mesenchymal stem cells promoted their differentiation into osteoblasts [47]. These studies showed that, during the process of osteogenic differentiation of ASCs, the DNA methylation levels of osteogenesis-specific genes Dlx5 and Runx2, and the CpG island region of the Osterix promoter were downregulated significantly, and the expression of these genes was upregulated. Seman et al. found that the DNA methylation level of the promoter region of the SLC30A8 gene in a diabetic population was higher than that in non-diabetic patients, which suggested that high DNA methylation of the SLC30A8 gene affects the occurrence of diabetes [50]. We observed that the DNA methylation levels and expression of DNMT genes in DOP-ASCs were upregulated significantly. After decreasing DNMTs by siRNA, we found that the expression of osteogenic differentiation factors RUNX2 and OPN was relatively increased, which indicated that DNA methylation had a close relationship with the osteogenic differentiation process of ASCs.

DNA methylation at specific sites is catalyzed by DNMTs, which might play various roles in cell differentiation. Dnmt3a, as the main methyltransferase in embryonic development and differentiation, is mainly located in the chromatin region and is highly expressed in oocytes, spermatogonia, and stem cells [51, 52]. Mark A. Casillas Jr NL et al. found that the expression of Dnmt3a was highly abundant in oocytes, but gradually decreased during maturation [53]. They observed that the overall methylation level of genomic DNA in senescent cells was reduced, which corresponded to the decrease in expression of Dnmt1, while some genes were hypermethylated with high expression of Dnmt3a and Dnmt3b [53]. Disturbances in epigenetic regulation may be a factor that contributes to diseases [54, 55]. In our study, RNA interference was used to silence the expression of Dnmt1, Dnmt3a, and Dnmt3b. Silencing of Dnmt3a promoted the expression of bone-related genes and Wnt/β-Catenin signaling pathway-related genes were induced, thereby promoting the osteogenic differentiation of DOP-ASCs. Overexpression of Dnmt3a by lentivirus infection confirmed that Dnmt3a significantly inhibited the expression of osteogenic-related genes and Wnt/β-Catenin signaling pathway in DOP-ASCs and the osteogenic differentiation ability of DOP-ASCs was restored after inhibition of Dnmt3a.

We also confirmed the osteogenic effects of Dnmt3a in DOP mice in vivo. BCP is considered to be a biomaterial with high porosity and penetration, which creates a favorable microenvironment for bone regeneration [56, 57]. Tang et al. implanted various BCP scaffolds into a critically sized bone defect model in OVX rats and applied Micro-CT to analyze new bone formation [58–60]. In our study, we seeded DOP-ASCs on BCP and implanted the scaffold into a mouse critically sized skull defect to assess the osteogenic capacity in vivo. Three-dimensional reconstruction of Micro-CT images showed that new bone formation in the Dnmt3a shRNA group had obviously increased compared with that in DOP-ASC and negative control groups. Furthermore, histology of the corresponding tissue samples was consistent with the results of Micro-CT, i.e., the amount of new bone formation in the Dnmt3a shRNA group was more obvious than that in DOP-blank and Negative Control.

DOP has become a severe public health problem. DNA methylation as a kind of stable epigenetic alteration is involved in bone formation and resorption [61]. Epigenetic modifications play an implant role in cell differentiation and development [62]. Many studies have demonstrated that DNA methylation is a therapeutic target for bone diseases [61]. Our study demonstrated that a high level of Dnmt3a may impair the osteogenic ability of ASCs, and the osteogenic differentiation ability of DOP-ASCs was restored after inhibition of Dnmt3a. Therefore, this study explains the decrease in the osteogenic capacity of DOP-ASCs from the viewpoint of epigenetics and provides a potential therapeutic target for the prevention and treatment of DOP.

## Conclusions

Our study showed that Wnt/β-catenin signaling pathway is a major player in the process of osteogenic differentiation of DOP-ASCs and DNA methylation is an important factor that affects the osteogenic differentiation of DOP-ASCs, which has significance for bone regeneration in DOP. Downregulation of Dnmt3a activated Wnt/β-catenin pathway, and promoted the osteogenic differentiation of DOP-ASCs. These findings indicate that Dnmt3a knockdown rescues the impaired osteogenic ability of DOP-ASCs *in vitro* and *in vivo*, thereby providing a possible approach for bone regeneration using DOP-ASCs in DOP patients.

## Data Availability

The datasets generated or analyzed during the current study can be obtained from the corresponding author in accordance with reasonable requirements.

## Abbreviations

DOP: Diabetic osteoporosis
DM: Diabetes mellitus
ASCs: Adipose-derived stem cells
PCR: Quantitative real-time polymerase chain reaction
ALP: Alkaline phosphatase
β-Catenin: Cadherin associated protein
Runx2: Runt-related transcription factor 2
Opn: Osteopontin
GAPDH: Glyceraldehyde phosphate dehydrogenase
DNMT1: DNA methyltransferases 1
DNMT3a: DNA methyltransferases 3a
DNMT3b: DNA methyltransferases 3b
BV/TV: Ratio of bone volume to tissue volume
BS/BV: The area of bone tissue per unit volume
TbTh: Trabecular thickness
